# In-Hospital Mortality of Sepsis Differs Depending on the Origin of Infection: An Investigation of Predisposing Factors

**DOI:** 10.3389/fmed.2022.915224

**Published:** 2022-07-13

**Authors:** Mark Pieroni, Ivan Olier, Sandra Ortega-Martorell, Brian W. Johnston, Ingeborg D. Welters

**Affiliations:** ^1^School of Computer Science and Mathematics, Liverpool John Moores University, Liverpool, United Kingdom; ^2^Liverpool Centre for Cardiovascular Science, Liverpool, United Kingdom; ^3^Institute of Life Course and Medical Sciences, University of Liverpool, Liverpool, United Kingdom; ^4^Liverpool University Hospitals National Health Service (NHS) Foundation Trust, Liverpool, United Kingdom

**Keywords:** sepsis, intensive care medicine, mortality risk, prognostic factors, origin of infection, logistic regression

## Abstract

Sepsis is a heterogeneous syndrome characterized by a variety of clinical features. Analysis of large clinical datasets may serve to define groups of sepsis with different risks of adverse outcomes. Clinical experience supports the concept that prognosis, treatment, severity, and time course of sepsis vary depending on the source of infection. We analyzed a large publicly available database to test this hypothesis. In addition, we developed prognostic models for the three main types of sepsis: pulmonary, urinary, and abdominal sepsis. We used logistic regression using routinely available clinical data for mortality prediction in each of these groups. The data was extracted from the eICU collaborative research database, a multi-center intensive care unit with over 200,000 admissions. Sepsis cohorts were defined using admission diagnosis codes. We used univariate and multivariate analyses to establish factors relevant for outcome prediction in all three cohorts of sepsis (pulmonary, urinary and abdominal). For logistic regression, input variables were automatically selected using a sequential forward search algorithm over 10 dataset instances. Receiver operator characteristics were generated for each model and compared with established prognostication tools (APACHE IV and SOFA). A total of 3,958 sepsis admissions were included in the analysis. Sepsis in-hospital mortality differed depending on the cause of infection: abdominal 18.93%, pulmonary 19.27%, and renal 12.81%. Higher average heart rate was associated with increased mortality risk. Increased average Mean Arterial Pressure (MAP) showed a reduced mortality risk across all sepsis groups. Results from the LR models found significant factors that were relevant for specific sepsis groups. Our models outperformed APACHE IV and SOFA scores with AUC between 0.63 and 0.74. Predictive power decreased over time, with the best results achieved for data extracted for the first 24 h of admission. Mortality varied significantly between the three sepsis groups. We also demonstrate that factors of importance show considerable heterogeneity depending on the source of infection. The factors influencing in-hospital mortality vary depending on the source of sepsis which may explain why most sepsis trials have failed to identify an effective treatment. The source of infection should be considered when considering mortality risk. Planning of sepsis treatment trials may benefit from risk stratification based on the source of infection.

## Introduction

Sepsis is defined as life-threatening organ dysfunction caused by a dysregulated host response to infection (1). It is not a uniform disease, but a complex syndrome of physiologic and biochemical abnormalities. Clinical experience supports the concept that prognosis, treatment, severity and time course vary depending on the source of infection (2, 3). Consequently, attempts have been made to characterize different types of sepsis based on clinical data, routine blood results and biomarkers (4). Mortality of sepsis ranges from 15% in patients with sepsis without shock to 56% in patients with sepsis with shock (5). However, mortality prediction for sepsis remains satisfactory at best (4).

Although numerous trials have been designed to explore treatment options for sepsis, so far, none of these has resulted in new therapies (6). A major shortcoming of many of these multi-center randomized clinical trials is the patient cohort investigated. Patients with sepsis manifest striking heterogeneity, not only with respect to the site or microbiology of the inciting infection but also with respect to the comorbid conditions present in the patient at the time of onset (7). Comorbidities, site of infection and pathogen factors impact the mortality attributed to sepsis. However, in most clinical trials differentiation between groups of sepsis is lacking and may have contributed to the negative outcome of these studies. Recently, attempts have been made to discriminate sub-phenotypes of sepsis based on panels of immunological markers. Although promising, these clinical phenotypes for sepsis (4) are complex, rely on measurement of biomarker profiles, and are thus not easy to implement into routine clinical applications.

Electronic health records are now commonly used to record all routine clinical data. This allows the construction of large databases, which not only structure and aggregate clinical data but also record outcome measures such as mortality, length of stay, and duration of ventilation. Alongside with routinely applied scoring systems such as the Acute Physiology and Chronic Health Evaluation (APACHE), the Sequential Organ Failure Assessment (SOFA), or the Simplified Acute Physiology Score (SAPS), novel outcome prediction models are being developed based on these large patient populations.

In this research, we investigate in-hospital mortality and predictors thereof in different cohorts of sepsis based on the origin of infection using data from the eICU Collaborative Research Database, a freely available multi-center database for critical care research (8). We hypothesize that mortality and factors influencing mortality risk differ between pulmonary, urinary, and abdominal sepsis as the three most relevant clinical presentations. We aim to identify unifying and distinct features in these groups. Comparisons will be made with established outcome prediction scores such as APACHE IV and SOFA to determine if more sophisticated models show superior performance in predicting hospital mortality in these different groups of septic patients.

## Materials and Methods

### Data Source

In this study, we used the eICU Collaborative Research Database (eICU) (8). The eICU is a multi-center intensive care unit (ICU) database with highly granular data for over 200,000 ICU admissions collected *via* eICU programs across the United States (US) (8). The eICU (V2.0) database comprises 200,859 ICU encounters for 139,369 unique patients admitted to hospitals between 2014 and 2015 to one of the 335 intensive care units across 208 hospitals in the US. All tables are deidentified to meet the safe harbor provisions of the US Health Insurance Portability and Accountability Act (HIPAA). This includes the removal of all protected health information and the assignment of random unique identifiers. The database includes demographic/hospital level records, vital signs and laboratory measurements, medications, APACHE components, care plan documentation, severity illness measures, diagnosis information, and treatment details.

### Data Extraction

**FIGURE 1:**
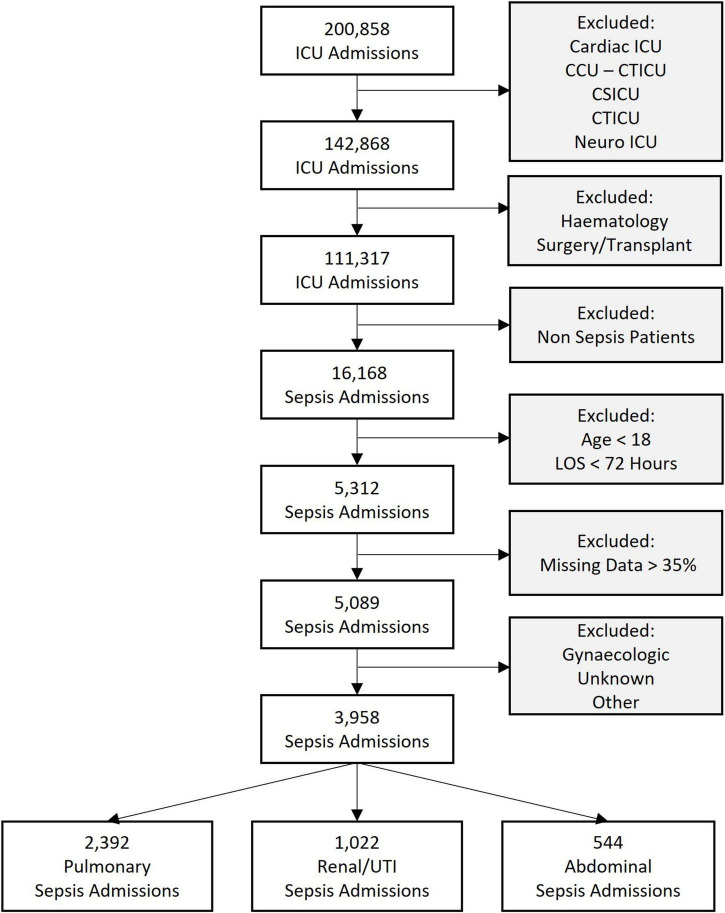
Flowchart of sepsis cohorts analyzed showing the inclusion and exclusion criteria. ICU, intensive care unit; CCU-CTICU, critical care unit-cardiothoracic intensive care unit, CSICU, cardio-surgical intensive care unit; LOS, length of stay; UTI, urinary tract infection.

We extracted data from the medical ICUs (MICU), surgical ICUs (SICU), and medical-surgical ICUs (Med-Surg ICU). Specialist critical care units such as cardiothoracic and cardio-surgical ICUs were excluded because of their specific patient cohorts with distinct presentations of sepsis. Patients after elective surgery and those with an underlying hematology diagnosis were also excluded, as their clinical presentation and course are distinct from patients with sepsis as the primary diagnosis. We then used the admission diagnosis codes, which are coded using the APACHE IV diagnosis system, to extract the admissions related to sepsis, and excluded patients < 18 years of age and with an ICU stay < 72 h. Lastly, all records with more than 35% missing data were excluded. These inclusion and exclusion criteria are represented in Figure 1.

We collected all electronic health record data from the acute phase of the ICU admission, defined as the first 72 h after admission. From this dataset, we excluded the first 6 h (resuscitation phase), where the priority is to stabilize the patient. Previous studies have used data from different time windows for outcome prediction, e.g., the first 24 h of the ICU admission (9). All dynamic features were organized into 1-h non-overlapping time series bins when extracting the data from the eICU database. This was to accommodate for different sampling frequencies of available data and the balance between missing data points and bin size. All time-varying variables were converted into tabular representations by extracting their means and standard deviations. The mean value of these time-varying variables, which represents the average of each time series, was named “Average” (Avg), e.g., the mean of the heart rate signal was coded as “Avg Heart Rate.” Similarly, the standard deviation, which is representing the variation in the time series, was coded as “Variations” (Var), e.g., Heart Rate Var.

### Outcome

The primary outcome was In-Hospital Mortality, which was coded as a binary variable to indicate whether the patient was dead (“1”) or alive (“0”).

### Study Aim

The aims of this study were (1). to define in-hospital mortality depending on the origin of infection and (2). To investigate predictors of in-hospital mortality for each of the most common types of sepsis: abdominal, urinary and chest sepsis.

### Definition of Sepsis Types

A cohort of patients with sepsis was extracted based upon the ICU admission diagnosis, which is coded using the APACHE IV diagnosis system (10) routinely recorded in the eICU database. From here, the following septic groups were identified: pulmonary, abdominal, and renal/urinary tract infection (UTI). Other smaller cohorts of septic patient groups were excluded either because of a lack of clarity regarding their clinical source (e.g., those encoded as “unknown” or “others”) or because of their considerably smaller number of cases (e.g., gynaecologic sepsis with less than 20 admissions). The prevalence in these groups was also reviewed against the encoded ICD codes for these patients to ensure that the relevant cohorts were well defined.

### Univariate Analysis

We used non-parametric statistical tests for continuous and categorical variables for univariate analysis of the three main groups of sepsis. The univariate analysis aims to compare variable distributions for significant differences amongst the sepsis groups. The Kruskal-Wallis test was applied to assess the differences among the sepsis groups for all continuous variables. Similarly, Pearson’s Chi-Square was used to assess differences for all categorical variables. *P*-values < 0.05 were considered statistically significant.

### Multiple Logistic Regression

Multiple logistic regression (LR) was used throughout the experiments. LR models the outcome probability or risk to be “1” (positive class) as $P\left(Y=1\right)=1/\left(1+\exp\left[-\sum_{k=0}^{K}\beta_{k}X_{k}\right]\right)$, where {β_0_,…,β_*K*_} are the model coefficients which are estimated by maximum likelihood (11). The LR coefficients are the logarithm odds ratios (OR) between the factors and the outcome. If a factor increased by one unit, its coefficient measures how much the outcome odd would increase or decrease, depending on whether the coefficient is positive or negative.

### Variable Selection and Cross-Validation

For LR, input variables were automatically selected using a sequential forward search algorithm over 10 dataset instances (10-fold cross-validation). For each iteration, an inner cycle of fivefold cross-validation was used to select relevant variables. Collectively this is referred to as nested cross-validation (Supplementary Figure 1). The selection algorithm starts with a baseline model (i.e., all coefficients but the intercept set to zero, β_*k*≠0_=0), and in each step, the variable which most improves the performance on the validation set is added (12).

### Model Performance

Model performance was measured using the area under the receiver operator characteristic (AUC) curve. AUC means and confidence intervals (CI) were calculated for each sepsis type.

### Model Explainability

To provide model explainability, we developed a forest plot for each sepsis type and a Sankey network diagram. The forest plots display the ORs and CIs associated with each clinical feature relevant to the developed LR models. The Sankey network diagram was used in a novel way to visualize the interactions between the significant clinical features and sepsis groups. For this, we selected the significant variables (*P* < 0.05) from the LR models (nodes on the left-hand side of the diagram) and generated links between them and the sepsis groups (nodes on the right-hand side of the diagram). Additionally, the absolute value of the OR interactions between clinical features and sepsis groups was represented by the height of the nodes, to provide further information regarding the relevance of each clinical feature.

### Comparisons of the Novel Models Against Established Critical Care Deterioration Scores

We compared the performance of two commonly used clinical scoring systems, the APACHE IV and SOFA score, which are typically used to predict in-hospital mortality for patients in critical care. We used the SOFA and APACHE IV scores as independent variables in a univariate LR model to produce the mortality risk estimate for the outcome. The purpose was to allow for a fair comparison between the developed models and the scores using the same methodology to evaluate how well each of them can predict the outcome.

The APACHE IV and SOFA scores are readily available in the eICU database. The APACHE IV scores were calculated based upon data collected on admission to the ICU, these values were available and listed in the eICU table “apachePatientResults.” Individual components of the SOFA score were calculated (13) for the first 3 days and then averaged. qSOFA scores were calculated by assigning points for (1). altered mental state (< 15 in the Glasgow Coma Scale), (2). Fast respiratory rate (> 22 breaths per minute) 3. Low blood pressure (systolic blood pressure < 100 mmHg).

## Results

### Sepsis Groups

A total of 3,958 ICU admissions were analyzed. A total of 2,393 patients were admitted with pulmonary sepsis, 1,044 with urinary sepsis and 544 with abdominal sepsis (Figure 1). Unadjusted statistical comparisons between the three sepsis groups are displayed in Table 1. Patients with urinary sepsis were older than patients with pulmonary and abdominal sepsis.

**TABLE 1:** Demographics, comorbidities, vital signs, and routine prognostic scores used for modeling.

	Abdominal (*N* = 544)	Pulmonary (*N* = 2,392)	Renal/UTI (*N* = 1,022)	*P*-value
**Outcome**				
In-hospital mortality	103 (18.9%)	461 (19.3%)	131 (12.8%)	< 0.001
**Demographics**				
Age	67.0 (56.0, 76.0)	67.0 (56.0, 77.0)	71.0 (60.0, 81.0)	< 0.001
Gender (Male)	276 (50.7%)	1,281 (53.6%)	437 (42.8%)	< 0.001
**Comorbidities**				
Myocardial infarction	45 (8.3%)	184 (7.7%)	85 (8.3%)	0.7862
CHF	85 (15.6%)	461 (19.3%)	204 (20.0%)	0.0932
PVD	27 (5.0%)	116 (4.8%)	53 (5.2%)	0.9172
Dementia	14 (2.6%)	166 (6.9%)	104 (10.2%)	< 0.001
COPD	81 (14.9%)	600 (25.1%)	136 (13.3%)	< 0.001
CTD	16 (2.9%)	70 (2.9%)	35 (3.4%)	0.7302
Peptic ulcer disease	14 (2.6%)	75 (3.1%)	35 (3.4%)	0.6552
Mild liver disease	31 (5.7%)	55 (2.3%)	26 (2.5%)	< 0.001
Uncomplicated DM	146 (26.8%)	713 (29.8%)	407 (39.8%)	< 0.001
Renal disease	94 (17.3%)	334 (14.0%)	165 (16.1%)	0.0712
Hemiplegia	45 (8.3%)	246 (10.3%)	146 (14.3%)	< 0.001
Severe liver disease	32 (5.9%)	49 (2.0%)	18 (1.8%)	< 0.001
Hypertension	269 (49.4%)	1,143 (47.8%)	564 (55.2%)	< 0.001
Hypothyroidism	16 (2.9%)	100 (4.2%)	43 (4.2%)	0.3882
Atrial fibrillation	70 (12.9%)	307 (12.8%)	144 (14.1%)	0.5962
Asthma	38 (7.0%)	219 (9.2%)	70 (6.8%)	0.0412
Seizures	32 (5.9%)	166 (6.9%)	83 (8.1%)	0.2312
Respiratory failure	10 (1.8%)	126 (5.3%)	46 (4.5%)	0.0032
CABG	25 (4.6%)	139 (5.8%)	46 (4.5%)	0.2142
Cancer	116 (21.3%)	422 (17.6%)	169 (16.5%)	0.0572
**Admission diagnosis**				
Pulmonary	181 (33.3%)	2,109 (88.2%)	350 (34.2%)	< 0.001
Cardiovascular	423 (77.8%)	1,788 (74.7%)	787 (77.0%)	0.1852
Infectious diseases	165 (30.3%)	569 (23.8%)	361 (35.3%)	< 0.001
Renal	205 (37.7%)	730 (30.5%)	662 (64.8%)	< 0.001
Gastrointestinal	323 (59.4%)	211 (8.8%)	91 (8.9%)	< 0.001
Oncology	20 (3.7%)	114 (4.8%)	24 (2.3%)	0.0042
Neurologic	85 (15.6%)	443 (18.5%)	270 (26.4%)	< 0.001
Endocrine	63 (11.6%)	330 (13.8%)	169 (16.5%)	0.0192
**Vitals**				
Avg heart rate	94.0 (81.9, 105.0)	90.2 (79.8, 100.8)	89.0 (78.1, 98.7)	< 0.001
Heart rate var	9.5 (6.9, 12.6)	10.0 (7.3, 13.5)	9.7 (7.1, 13.4)	0.0201
Avg SaO_2_	96.6 (95.3, 98.2)	96.6 (95.1, 98.0)	97.1 (95.9, 98.5)	< 0.001
SaO_2_ var	1.9 (1.4, 2.5)	2.1 (1.6, 2.7)	1.8 (1.3, 2.5)	< 0.001
Avg GCS total	13.8 (10.5, 14.9)	11.3 (9.0, 14.3)	13.6 (10.0, 14.8)	< 0.001
GCS total var	0.7 (0.2, 1.7)	0.9 (0.4, 1.9)	0.6 (0.3, 1.5)	< 0.001
Avg respiratory rate	20.5 (18.0, 23.9)	21.4 (18.6, 24.8)	20.1 (17.6, 23.3)	< 0.001
Respiratory rate var	3.8 (2.9, 5.0)	4.0 (2.9, 5.2)	3.7 (2.9, 4.9)	0.0171
**Vitals**				
Avg temperature °C	36.8 (36.6, 37.2)	36.9 (36.6, 37.2)	36.8 (36.6, 37.2)	0.0431
Temperature °C var	0.4 (0.3, 0.6)	0.4 (0.3, 0.6)	0.4 (0.3, 0.6)	0.0411
Avg MAP	76.8 (72.4, 84.3)	80.1 (74.4, 87.6)	78.6 (73.2, 86.4)	< 0.001
MAP var	9.1 (7.3, 11.6)	9.6 (7.5, 12.1)	9.9 (7.9, 12.5)	< 0.001
Avg WBC	13.5 (9.3, 19.1)	12.2 (8.6, 16.9)	12.6 (8.7, 18.0)	< 0.001
WBC var	2.5 (1.3, 4.5)	2.0 (1.1, 3.6)	2.3 (1.1, 4.2)	< 0.001
Avg albumin	2.3 (1.9, 2.6)	2.3 (2.0, 2.7)	2.3 (2.0, 2.7)	0.0161
Albumin var	0.2 (0.1, 0.3)	0.1 (0.1, 0.3)	0.1 (0.1, 0.2)	0.0041
Avg platelets	163.8 (100.7, 239.8)	180.0 (124.0, 249.0)	164.6 (106.0, 230.8)	< 0.001
Platelets var	21.2 (12.0, 37.1)	18.6 (9.2, 31.8)	17.7 (9.2, 29.8)	0.0071
Avg PaO_2_	92.4 (75.9, 115.4)	91.0 (75.8, 113.1)	97.0 (79.4, 120.0)	0.0181
PaO_2_ var	20.6 (11.2, 43.4)	20.6 (11.1, 37.8)	19.3 (9.2, 34.3)	0.3321
Avg PaCO_2_	36.3 (31.6, 42.0)	39.3 (34.0, 46.3)	35.8 (30.2, 41.2)	< 0.001
PaCO_2_ var	4.1 (2.6, 6.4)	4.0 (2.2, 7.1)	3.5 (2.1, 5.8)	0.0821
Avg FiO_2_	43.0 (35.0, 60.0)	50.0 (40.0, 70.0)	40.0 (33.3, 53.6)	< 0.001
FiO_2_ Var	7.5 (0.0, 17.9)	9.5 (3.5, 18.3)	7.1 (0.7, 15.2)	0.1121
Avg total bilirubin	0.9 (0.5, 2.3)	0.6 (0.4, 1.0)	0.6 (0.4, 1.2)	< 0.001
Total bilirubin var	0.2 (0.1, 0.5)	0.1 (0.1, 0.3)	0.1 (0.1, 0.3)	< 0.001
Avg creatinine	1.4 (0.9, 2.6)	1.0 (0.7, 1.8)	1.4 (0.9, 2.3)	< 0.001
Creatinine var	0.2 (0.1, 0.4)	0.1 (0.1, 0.3)	0.2 (0.1, 0.4)	< 0.001
Avg BUN	29.6 (17.7, 49.8)	25.5 (16.0, 41.0)	31.0 (18.3, 48.9)	< 0.001
BUN var	4.8 (2.4, 8.8)	4.0 (2.1, 7.3)	4.2 (2.1, 8.6)	< 0.001
Avg PH	7.4 (7.3, 7.4)	7.4 (7.3, 7.4)	7.4 (7.3, 7.4)	< 0.001
pH Var	0.0 (0.0, 0.1)	0.0 (0.0, 0.1)	0.0 (0.0, 0.1)	0.0681
Avg sodium	139.0 (136.0, 142.7)	139.8 (136.7, 143.0)	140.0 (136.9, 144.0)	< 0.001
Sodium var	1.8 (1.2, 3.1)	1.9 (1.2, 2.8)	2.1 (1.3, 3.1)	0.0171
Avg glucose	130.8 (110.0, 161.5)	141.0 (114.6, 170.6)	139.2 (115.8, 170.5)	< 0.001
Glucose var	24.7 (15.3, 37.7)	26.8 (17.1, 41.4)	29.8 (19.5, 45.8)	< 0.001
Avg hematocrit	28.9 (25.6, 32.8)	29.9 (26.5, 34.0)	29.5 (26.5, 33.3)	< 0.001
Hematocrit var	2.1 (1.2, 3.1)	1.6 (0.9, 2.6)	1.5 (0.9, 2.5)	< 0.001
Avg urine	161.1 (68.8, 364.1)	226.2 (96.3, 475.0)	224.2 (91.4, 551.0)	< 0.001
Urine var	70.6 (33.3, 158.9)	108.9 (54.5, 208.9)	106.1 (50.3, 226.3)	< 0.001
**Respiration**				
Intubated	289 (53.1%)	1,914 (80.0%)	486 (47.6%)	< 0.001
**Drugs**				
Norepinephrine	241 (44.3%)	861 (36.0%)	436 (42.7%)	< 0.001
Vasopressin	80 (14.7%)	225 (9.4%)	111 (10.9%)	0.0012
Phenylephrine	56 (10.3%)	147 (6.1%)	60 (5.9%)	0.0012
Dopamine	18 (3.3%)	60 (2.5%)	44 (4.3%)	0.0202
Epinephrine	15 (2.8%)	36 (1.5%)	15 (1.5%)	0.1022
Dobutamine	16 (2.9%)	43 (1.8%)	24 (2.3%)	0.1972
**Scores**				
Charlson CI	2.0 (0.0, 3.0)	2.0 (0.0, 3.0)	2.0 (1.0, 3.0)	0.4031
SOFA	4.0 (1.0, 7.0)	4.0 (2.0, 7.0)	3.0 (1.0, 6.0)	< 0.001
APACHE IV	73.0 (61.0, 88.0)	73.0 (58.0, 89.0)	73.0 (62.0, 87.0)	0.8951
SIRS	2.0 (1.0, 2.0)	2.0 (1.0, 2.0)	1.0 (1.0, 2.0)	< 0.001
qSOFA	1.0 (1.0, 2.0)	1.0 (1.0, 2.0)	1.0 (1.0, 2.0)	< 0.001
**Unit stay type**				< 0.001
Admit	453 (83.3%)	1,991 (83.2%)	863 (84.4%)	
Other/Stepdown/Transfer	67 (12.3%)	277 (11.6%)	138 (13.5%)	
Readmit	24 (4.4%)	124 (5.2%)	21 (2.1%)	
**Unit type**				< 0.001
Med-surg ICU	386 (71.0%)	1,830 (76.5%)	785 (76.8%)	
MICU	104 (19.1%)	440 (18.4%)	197 (19.3%)	
SICU	54 (9.9%)	122 (5.1%)	40 (3.9%)	
**Admission duration**				
Hospital LOS	287.3 (190.7, 470.7)	264.4 (172.7, 400.2)	222.7 (159.6, 343.7)	< 0.001
ICU LOS	125.9 (92.1, 209.0)	140.7 (97.7, 228.8)	112.1 (87.5, 159.3)	< 0.001

*The first column displays the data characteristics (variables). Columns second to fourth show summary statistics of all the variables for each sepsis group. Sepsis group cohort sizes are reported under the group name. Numeric variables are reported with the median and IQR (in parentheses), while categorical variables are reported with the frequency and proportion (in parenthesis). The resulting statistical tests are reported in the fifth column in the form of p-values. Any p-value smaller than 0.001 was indicated as “< 0.001.” CHF, congestive heart failure; PVD, Peripheral vascular disease; COPD, Chronic obstructive pulmonary disease; CTD, Connective tissue diseases; DM, diabetes mellitus; CABG, Coronary artery bypass graft surgery; SaO_2_, oxygen saturation; GCS, Glasgow coma scale; MAP, Mean Arterial Pressure; WBC, white blood cells count; PaO_2_, partial pressure of oxygen; FiO_2_, Fraction of Inspired Oxygen; BUN, blood urea nitrogen; SOFA, Sequential Organ Failure Assessment; qSOFA, quick SOFA; APACHE, Acute Physiology And Chronic Health Evaluation; SIRS, Systemic Inflammatory Response Syndrome; ICU, Intensive Care Unit; Med-Surg ICU, medical-surgical ICU; MICU, medical ICU, SICU, surgical ICU; LOS, length of stay; Avg, average (mean); Var, variation (standard deviation).*

With the exception of hypertension, there were no significant differences in cardiovascular comorbidities between the groups. We found group differences that were statistically significant (*p*-value < 0.05) for comorbidities such as mild and severe liver disease, dementia and respiratory diseases (COPD, asthma). We also observed significant group differences in vital signs (average heart rate, average mean arterial pressure (MAP), average saturation, average respiratory rate and average temperature) and blood counts (average lymphocyte count, average white blood cell count, average platelet count, and hematocrit). Blood gas results differed between groups with regards to average pH, average pO_2_ and average pCO_2_. Liver and kidney function was also significantly different between groups. Compared to patients with pulmonary or abdominal sepsis, a smaller proportion of patients with urinary sepsis required inotropes during their stay.

While there was a significant difference between SOFA and qSOFA scores between the groups, Charlson comorbidity index and APACHE IV score were comparable between abdominal, urinary and pulmonary sepsis.

### Evaluation of Model Performances

**FIGURE 2:**
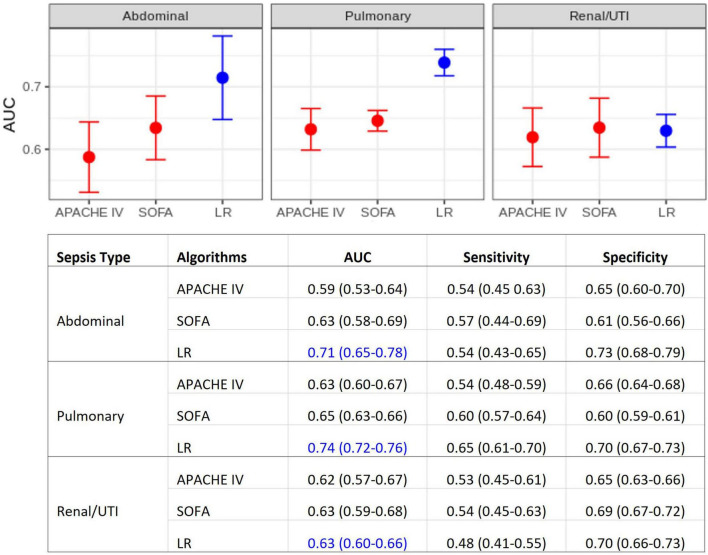
Model performance comparisons. (Top) Area under the ROC curve (AUC) for each sepsis group. Average AUC (filled circles) and confidence intervals (vertical bars) estimated after the 10 repetitions of the outer cross-validation. Deterioration scores (APACHE IV and SOFA) models are represented in red, LR models in blue. (Bottom) Detailed comparison, also including sensitivity and specificity. APACHE IV, Acute Physiology And Chronic Health Evaluation IV; SOFA, Sequential Organ Failure Assessment; LR, multiple logistic regression.

Figure 2 displays the results of the comparison between the developed multivariate models and the APACHE IV and SOFA scores. These AUC results show that, for pulmonary and abdominal sepsis, the novel models outperformed APACHE IV and SOFA scores (AUC 0.74 and 0.71, respectively), but were not superior in urinary sepsis (AUC 0.63).

**FIGURE 3:**
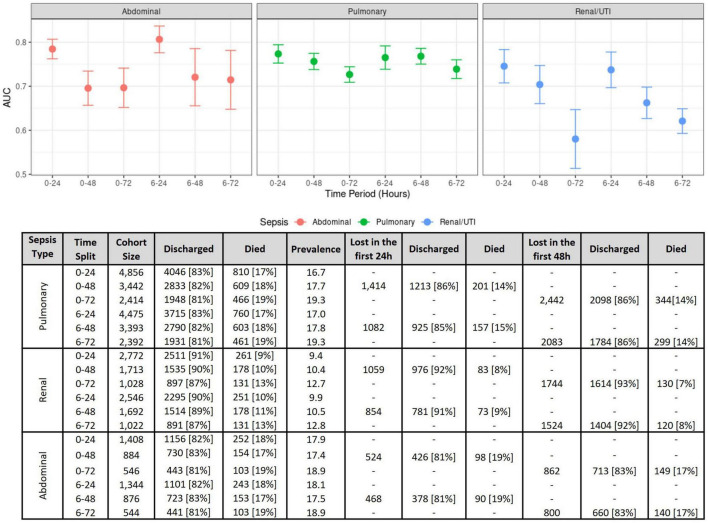
Model performance measures on several time windows. (Top) Model performance comparisons as measured using the AUC for each sepsis group at several time intervals. The figure shows AUC means and confidence intervals estimated after the 10 repetitions of the outer cross-validation with logistic regression. (Bottom) Effects of different time windows on cohort size and mortality rates.

Comparisons using different time windows for data extraction was performed to assess (a) how this decision impacts model performances, and (b) how our analysis compares to previous studies. Figure 3 compiles the results obtained for the first 24, 48, and 72 h, with or without the inclusion of the first 6 h. The best results were obtained when using the first 24 h, where the cohort sizes were generally twice the size of those at 72 h (see the bottom of Figure 3), as a great proportion of patients either died or were discharged between 24 and 72 h after ICU admission.

### Explanatory Analysis

**FIGURE 4:**
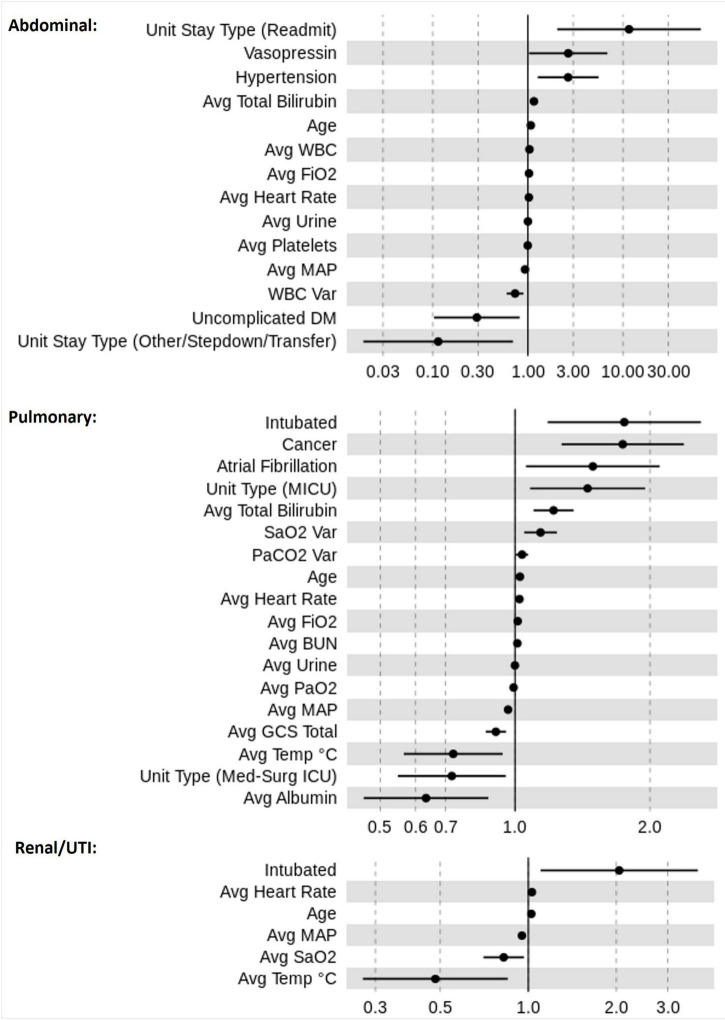
Odds ratio (OR) estimates for LR. The figure displays the pooled ORs average (filled circles) and confidence intervals (vertical bars) for all significant features (*p* < 0.05) selected by the feature selection algorithms for the sepsis groups: pulmonary, abdominal, and renal/UTI. An OR of 1 represents a baseline risk, with values < 1 indicating a reduction in risk for the outcome, and > 1 indicating an increased risk in relation to the outcome.

Figure 4 displays the ORs for risk factors in the three sepsis groups as estimated across the 10 dataset instances. Higher age and higher average heart rate were associated with increased mortality risk. Increased values in average MAP were associated with a reduced mortality risk across all sepsis groups. Our LR models identified significant factors that were relevant only for certain sepsis groups. For instance, atrial fibrillation and cancer were associated with an increased mortality risk only in pulmonary sepsis, but not in urinary or abdominal sepsis. Contrastingly, in abdominal sepsis hypertension represented a relevant risk factor of mortality. Interestingly, abdominal sepsis was the only group for which uncomplicated diabetes represented a significant protective factor regarding mortality risk.

A number of factors were relevant to more than one sepsis group. For instance, the most influential factor for increased mortality risk was “intubation” for urinary and pulmonary sepsis groups, however, in abdominal sepsis “readmitted to ICU” represented the most important factor. A rise in risk was associated with higher “average FiO_2_” and “average total bilirubin” values in both abdominal and pulmonary sepsis, but not in urinary sepsis. Distinctively, in pulmonary and renal sepsis lower average temperature was indicative of reduced mortality risk. The average albumin was associated with the greatest risk reduction in pulmonary sepsis, whereas in renal and abdominal sepsis “average temperature” and “unit stay type (other/stepdown/transfer)” represented important variables.

Moreover, results illustrated that the average value for certain parameters was relevant while for other variables, the average variation played a greater role in mortality risk prediction. For instance, mortality risk reduces in renal sepsis when there is an increase in “average SaO_2_.” This is dissimilar to pulmonary sepsis, for which higher “SaO_2_ variation” increased the risk of mortality.

**FIGURE 5:**
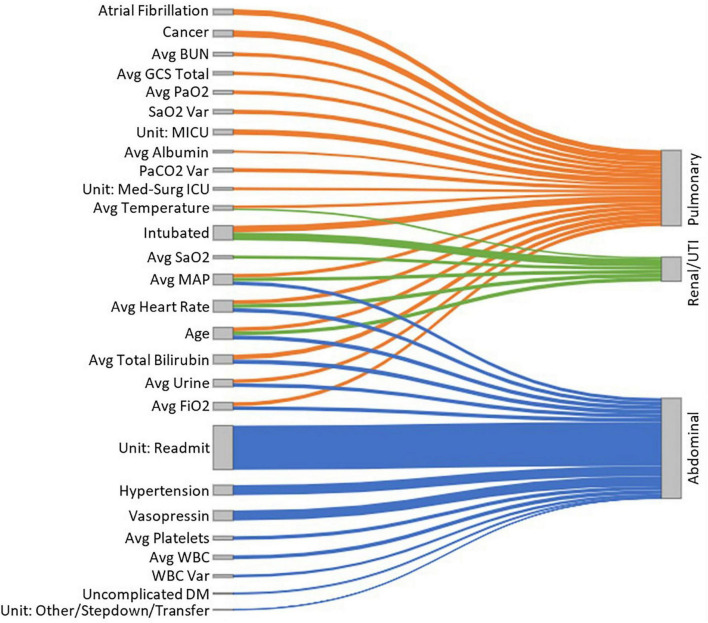
A Sankey diagram representing the relationship between several clinical features (nodes on the left-hand side) and the sepsis groups (nodes on the right-hand side), with the link widths representing the absolute ORs proportional to the risk of in-hospital mortality for each of the sepsis groups.

Figure 5 presents a Sankey network diagram displaying the relationship between several clinical features and the sepsis groups. It shows that “intubation,” “average total bilirubin,” “average FiO_2_,” “average urine output,” “average heart rate” and “average MAP” had the greatest overlap between sepsis groups. In abdominal sepsis, readmission had the greatest influence on the risk of in-hospital mortality compared to any other variables included in the model.

## Discussion

In this study, we conduct a LR analysis of several types of sepsis based on the origin of infection. Our results showed that using LR as a relatively simple approach to ML was sufficient to obtain good to very good models for renal, abdominal and pulmonary sepsis that consistently outperformed the established risk scores for predicting in-hospital mortality. Biomedical and social scientists are usually familiar with the results provided by LR models, hence their great popularity. The major drawbacks of LR are the linearity and normality assumptions of the data which could yield biased models.

Traditionally, outcome prediction in sepsis is based on clinical scores, such as SOFA, APACHE, or SAPS. Such mortality prediction scores for critically ill patients are used worldwide and have been extensively validated (14). These models, however, may not be ideal for routine clinical use as they lack granularity and are designed for use at ICU admission, thus neglecting the change of physiological parameters over time. So far, only a limited number of studies describe prognostication for in-hospital mortality in patients with sepsis comparing different sources of infection as an independent factor (15). In this study, we address this knowledge gap by (a) comparing different risk factors for each sepsis type and (b) highlighting specific factors associated with in-hospital mortality in the distinct sepsis groups, depending on the origin of the underlying infection. This approach may help to address the heterogeneity of the patient population with sepsis, to define discrete patient populations to guide the development of effective therapies and identify cohorts that benefit from certain interventions.

A fundamental difference between our models and existing ones for outcome prediction is that we include data from a longer observation period. For frequently measured variables such as vital signs, up to 72-h’ worth of data points were used, with measurements recorded every hour. We extracted the mean and the standard deviation of all data points available to factor in change over time, with the former indicating the average values for each patient, and the latter indicating the range of variation in those values, e.g., a high heart rate variation may be indicative of some form of hemodynamic instability. However, the mean and the standard deviation represent a crude representation of change over time, and further research is required to investigate and define the best mathematical approach to reflect the variation of variables, particularly those with frequent measurement, e.g., heart rate or blood pressure.

We performed outcome prediction at various time points during the early phase of sepsis. Our results demonstrate that the performance of ML models drops over the first 72 h after ICU admission in all the types of sepsis studied. Model performance is best maintained in pulmonary sepsis, while loss of performance is greatest in urinary sepsis. A possible explanation is that the causes of death from sepsis vary over time. While early deaths occur in about a third of septic patients and are mainly attributable to multiple organ failure caused by the primary infection, late deaths are influenced by end-of-life decisions and often relate to recurrent or late infections (16).

Early deaths in sepsis are typically associated with a hyperinflammatory “cytokine storm” response with fever, refractory shock, acidosis, and hypercatabolism (17). If regulation of the immune response from hyperinflammation to normal activity fails after the acute phase, patients enter a marked immunosuppressive state. Later deaths after the acute phase occur due to an inability to clear primary infections and the development of secondary infections (17). Taken into account the biphasic or even polyphasic course of sepsis, mortality prediction in the acute phase will differ from models predicting later mortality. Hence models that only include admission data are likely to disproportionately focus on early death occurring in the first 24 h of admission. Whilst optimizing data collection periods may improve outcome prediction, the ideal model should reflect dynamic changes and risk profile throughout the Intensive Care admission.

Our results indicate that prediction after the acute phase of sepsis is more complex and not well described in existing prognostication models. In addition, outcome prognostication is often performed early during the ICU stay, and many scores such as APACHE IV, are only validated for use on admission to Critical Care. Generation of a logistic regression model to predict mortality represents the first step in producing a score for wider clinical use; comparison to existing models is required to justify progression to external validation, refinement and eventually development of a new score with different weighting of individual risk factors. The degree of organ failure associated with the type of sepsis and the early progression of disease varies between sepsis groups and may be influenced differently in each group by early deaths and vice versa, early recovery and discharge alive. This assumption is supported by the higher dropout of cases in the urinary sepsis group compared to other sepsis caused by abdominal and chest infections.

Sepsis is not a uniform disease, but a syndrome characterized by the striking variation of biological features (18). Systematic analysis of these features, using data mining, and advanced statistical methods or machine learning, may allow the identification of types of sepsis with different risk profiles and responses to treatment. In an attempt to classify different types of sepsis, several approaches have been chosen (19). More sophisticated definitions of distinct molecular endotypes are based on leukocyte genome-wide expression profiles from samples collected on ICU admission (20–22). However, the implementation of these complex prognostic and predictive strategies at the bedside of patients is limited (23) due to the need for expensive laboratory analysis, which is not routinely available and is often too time-consuming to allow clinical decision making. Different statistical methods, including latent class analysis (24, 25), group-based trajectory modeling (26) and various machine learning algorithms (27) have been applied to large clinical data sets.

Clinicians instantaneously recognize that bacterial sepsis in young otherwise healthy patients carries a better prognosis than fungal sepsis in an elderly hematology patient. Similarly, urinary sepsis is commonly perceived as less fatal than chest or intraabdominal sepsis. A systematic review which addressed the impact of the source of infection on mortality (28), identified several studies in which lower in-hospital mortality was observed for urinary sepsis compared to respiratory sepsis. This observation was independent of the stage of sepsis with lower mortality observed in sepsis, severe sepsis and septic shock. Our results confirm the observation that in-hospital mortality is lower in critically ill patients with urinary sepsis compared to abdominal and respiratory sepsis. Factors influencing mortality differed between sepsis groups in our research, e g., ICU readmission was a significant risk factor in abdominal sepsis, but played no role in pulmonary or urinary sepsis, indicating that the numerous ICU stays required for complex abdominal sepsis are associated with a worsening prognosis. In contrast, for pulmonary and urinary sepsis, the need for invasive ventilation was a significant risk factor for mortality. The origin of infection is often known to treating physicians early in the clinical course and as such, outcome prediction based on the type of causative infection using clinical data only, may be easier to implement than models relying on complex combinations of clinical data and biomarkers, which are often not readily available at the bedside. Modern monitoring devices allow the integration of such prognostic algorithms into their software package and facilitate easy clinical implementation for all patients requiring regular monitoring.

The strength of this study is that we used the eICU database, a public database containing a large number of datasets for critically ill patients to generate our models. Moreover, we included time series for vital signs and laboratory tests for up to 72 h after admission to ICU in patients with different origins of sepsis and demonstrated that the models outperformed existing prediction tools. However, our study also has limitations. External validation and comparison with other machine learning approaches are required to explore the transferability and generalizability of our models in different critical care settings. Furthermore, the combination of molecular diagnostics such as transcriptomics and genomics with the routinely available clinical data used in our model may further improve the performance.

## Conclusion

We present a logistic regression model for different types of sepsis which are defined by their origin of infection using routinely available clinical data from a large publicly available dataset. We demonstrate that factors of importance show considerable heterogeneity depending on the source of infection.

## Data Availability Statement

Publicly available datasets were analyzed in this study. This study can be found in the eICU Collaborative Research Database repository (https://eicu-crd.mit.edu/).

## Ethics Statement

Ethical review and approval was not required for the study on human participants in accordance with the local legislation and institutional requirements. Written informed consent for participation was not required for this study in accordance with the national legislation and the institutional requirements.

## Author Contributions

MP extracted the data. MP and IO implemented the code and analyzed the data. IW and MP agreed to be the guarantors of all aspects of the work. All authors made substantial contributions to the design of the study, interpreted the results, drafted and critically revised the manuscript, and approved the final version and were involved in the study design and the selection of relevant variables from the dataset.

## Conflict of Interest

The authors declare that the research was conducted in the absence of any commercial or financial relationships that could be construed as a potential conflict of interest.

## Publisher’s Note

All claims expressed in this article are solely those of the authors and do not necessarily represent those of their affiliated organizations, or those of the publisher, the editors and the reviewers. Any product that may be evaluated in this article, or claim that may be made by its manufacturer, is not guaranteed or endorsed by the publisher.
