# Supplementary material for: In-Hospital Mortality of Sepsis Differs Depending on the Origin of Infection: An Investigation of Predisposing Factors
Source: Front Med (Lausanne). 2022 Jul 13;9:915224. doi: 10.3389/fmed.2022.915224 (PMC9326002; doi:10.3389/fmed.2022.915224)
Supplement: Supplementary file 1 [file Data_Sheet_1.PDF]

# Supplementary Material

Table S1: All variables used in each sepsis group for the final developed model

| Sepsis Groups | All Features Selected for Logistic Regression |
|---|---|
| **Abdominal** | Age, Albumin Var, Asthma, Atrial Fibrillation, Avg Albumin, Avg Creatinine, Avg FiO2, Avg GCS Total, Avg Glucose, Avg Heart Rate, Avg Hematocrit, Avg Lymphs, Avg MAP, Avg PaCO2, Avg PaO2, Avg PH, Avg Platelets, Avg Resp Rate, Avg Sodium, Avg Temp °C, Avg Total Bilirubin, Avg Urine, Avg WBC, BUN Var, CABG, Cancer, Creatinine Var, CTD, Dementia, Dobutamine, Dopamine, Endocrine, FiO2 Var, GCS Total Var, Gender (Male), Glucose Var, Heart Rate Var, Hematocrit Var, Hemiplegia, Hypertension, Hypothyroidism, Infectious Diseases, Intubated, Lymphs Var, MAP Var, Mild Liver Disease, Neurologic, Norepinephrine, Oncology, PaCO2 Var, PaO2 Var, Phenylephrine, Platelets Var, Pulmonary, Renal Disease, Resp Rate Var, Respiratory Failure, SaO2 Var, Seizures, Severe Liver Disease, Sodium Var, Temp °C Var, Uncomplicated DM, Unit Stay Type (Admit), Unit Stay Type (Other/Stepdown/Transfer), Unit Stay Type (Readmit), Unit Type (SICU), Urine Var, Vasopressin, WBC Var. |
| **Pulmonary** | Age, Atrial Fibrillation, Avg Albumin, Avg BUN, Avg FiO2, Avg GCS Total, Avg Heart Rate, Avg MAP, Avg PaO2. Avg SaO2. Avg Temp °C, Avg Total Bilirubin, Avg Urine, Cancer, Cardiovascular, CHF, Dementia, GCS Total Var, Gender (Male), Heart Rate Var, Hypothyroidism, Intubated, Norepinephrine, Oncology, PaCO2 Var, Phenylephrine, Platelets Var, Renal Disease, Resp Rate Var, Respiratory Failure, SaO2 Var, Total Bilirubin Var, Unit Stay Type (Admit), Unit Stay Type (Readmit), Unit Type (Med-Surg ICU), Unit Type (MICU), Vasopressin. |
| **Renal/UTI** | Age, Albumin Var, Asthma, Atrial Fibrillation, Avg Albumin, Avg BUN, Avg Creatinine, Avg FiO2, Avg GCS Total, Avg Glucose, Avg Heart Rate, Avg Hematocrit, Avg Lymphs, Avg MAP, Avg PaCO2, Avg PaO2, Avg PH, Avg Platelets, Avg Resp Rate, Avg SaO2, Avg Sodium, Avg Temp °C, Avg Total Bilirubin, Avg Urine, Avg WBC, BUN Var, CABG, Cancer, Cardiovascular, CHF, COPD, Creatinine Var, CTD, Dementia, Dobutamine, Dopamine, Endocrine, Epinephrine, FiO2 Var, Gastrointestinal, GCS Total Var, Gender (Male), Glucose Var, Heart Rate Var, Hematocrit Var, Hemiplegia, Hypertension, Hypothyroidism, Infectious Diseases, Intubated, Lymphs Var, MAP Var, Mild Liver Disease, Myocardial Infarction, Neurologic, Norepinephrine, Oncology, PaCO2 Var, PaO2 Var, Peptic Ulcer Disease, PH Var, Phenylephrine, Platelets Var, Pulmonary, PVD, Renal, Renal Disease, Resp Rate Var, Respiratory Failure, SaO2 Var, Seizures, Severe Liver Disease, Sodium Var, Temp °C Var, Total Bilirubin Var, Uncomplicated DM, Unit Stay Type (Admit), Unit Stay Type Other/Stepdown/Transfer), Unit Stay Type (Readmit),Unit Type (Med-Surg ICU), Unit Type (MICU), Unit Type (SICU), Urine Var, Vasopressin, WBC Var. |

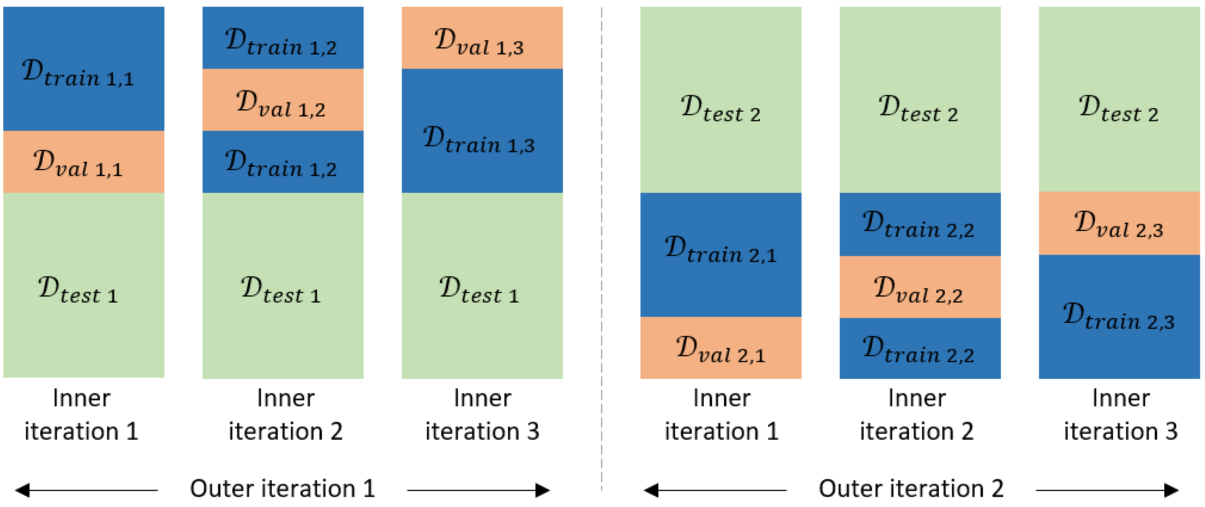


**Figure S1:** The example displays a 2x3-fold nested cross-validation. $D_{train}$, $D_{val}$ and $D_{test}$ represent a proportion of the training, validation and test data used in each iteration.

Nested cross-validation

Nested cross-validation is commonly used to train a model in which hyperparameters also need to be optimised. In our case, the implementation of a forward sequential search algorithm for feature selection. In each fold of the outer cross-validation, the hyperparameters of the model are tuned independently to minimise an inner cross-validation estimate of the performance. This eliminates the bias introduced by the inner cross-validation procedure as the test data in each iteration of the outer cross-validation has not been used to optimise the performance of the model in any way, and may, therefore, provide a more reliable criterion for selecting the best model [1].


[1] J. Wainer and G. Cawley, 'Nested cross-validation when selecting classifiers is overzealous for most practical applications', *Expert Syst. Appl.*, vol. 182, p. 115222, Nov. 2021, doi: 10.1016/J.ESWA.2021.115222.
